# Strategies for engineering advanced nanomedicines for gas therapy of cancer

**DOI:** 10.1093/nsr/nwaa034

**Published:** 2020-02-27

**Authors:** Yingshuai Wang, Tian Yang, Qianjun He

**Affiliations:** Guangdong Provincial Key Laboratory of Biomedical Measurements and Ultrasound Imaging, National-Regional Key Technology Engineering Laboratory for Medical Ultrasound, School of Biomedical Engineering, Health Science Center, Shenzhen University, Shenzhen 518060, China; Guangdong Provincial Key Laboratory of Biomedical Measurements and Ultrasound Imaging, National-Regional Key Technology Engineering Laboratory for Medical Ultrasound, School of Biomedical Engineering, Health Science Center, Shenzhen University, Shenzhen 518060, China; Guangdong Provincial Key Laboratory of Biomedical Measurements and Ultrasound Imaging, National-Regional Key Technology Engineering Laboratory for Medical Ultrasound, School of Biomedical Engineering, Health Science Center, Shenzhen University, Shenzhen 518060, China

**Keywords:** nanomedicine, gas therapy, drug delivery, controlled release, cancer treatment

## Abstract

As an emerging and promising treatment method, gas therapy has attracted more and more attention for treatment of inflammation-related diseases, especially cancer. However, therapeutic/therapy-assisted gases (NO, CO, H_2_S, H_2_, O_2_, SO_2_ and CO_2_) and most of their prodrugs lack the abilities of active intratumoral accumulation and controlled gas release, resulting in limited cancer therapy efficacy and potential side effects. Therefore, development of nanomedicines to realize tumor-targeted and controlled release of therapeutic/therapy-assisted gases is greatly desired, and also the combination of other therapeutic modes with gas therapy by multifunctional nanocarrier platforms can augment cancer therapy efficacy and also reduce their side effects. The design of nanomedicines with these functions is vitally important, but challenging. In this review, we summarize a series of engineering strategies for construction of advanced gas-releasing nanomedicines from four aspects: (1) stimuli-responsive strategies for controlled gas release; (2) catalytic strategies for controlled gas release; (3) tumor-targeted gas delivery strategies; (4) multi-model combination strategies based on gas therapy. Moreover, we highlight current issues and gaps in knowledge, and envisage current trends and future prospects of advanced nanomedicines for gas therapy of cancer. This review aims to inspire and guide the engineering of advanced gas-releasing nanomedicines.

## INTRODUCTION

Cancer is a major disease endangering human health because of its high heterogeneity and complexity. Simple elimination of cancer cells with cytotoxic radio-/chemo-therapeutic drugs is often not very efficient, and can even be adverse because of stimulated drug resistance, metastasis and recurrence. In recent years, with advances in knowledge of cancer, the tumor microenvironment (TME) has been discovered to be of vital importance for occurrence and evolution of tumors. The TME has some unique pathological characteristics including hypoxia, high reducibility, slight acidity, over-expression of hydrogen peroxide and increased vascular permeability, mainly resulting from the rapid energy metabolism of cancer cells. Signaling molecules in the TME have indispensable roles in the communication among various cancer-associated cells, which ensures the orderly running of cancer growth, proliferation, resistance and metastasis. Functional damage to one or two TME factors often causes compensatory self-repair or even resistance and circumvention, but the interdiction of signalling pathways can efficiently destroy the TME [1].

Among signalling molecules, some endogenous gasotransmitters including NO, CO and H_2_S have important roles in promoting growth, proliferation and metastasis of cancer-associated cells in the TME [2]. A small amount of endogenous gasotransmitter can regulate vasodilatation, neurotransmission, anti-inflammatory and antioxidative reactions. The commonly accepted mechanism is that NO, CO and H_2_S gasotransmitters solidly bind to haem iron centres in various proteins, especially haemoglobin in mitochondria, to regulate cellular bioenergetics (Table 1) with a ‘Janus-faced’ pharmacological character [3]. Low concentrations (below nM level) of these gases in the TME mediate antioxidant, signalling and positive bioenergetic mechanisms for protection of cancer cells in favour of tumor cell proliferation, growth and metastasis, while high concentrations (at or above nM level) of these gases are toxic to cancer cells by inhibiting mitochondrial respiratory metabolism. Both complete inhibition of their expressions in the TME and distinct enhancement of their concentrations by stimulation or delivery will seriously affect the behaviours of cancer cells, including impairment of cell protection and self-repair functions, and quick exhaustion of cancer cell energy. In addition, these gasotransmitters can sensitize and enhance other traditional therapies, and can also reduce the toxic side effects of traditional therapies by protecting normal cells from non-specific damage [4]. Such a selective cancer-killing and normal-protecting effect is highly desirable, and the application of these therapeutic gases for cancer treatment (termed ‘gas therapy of cancer’) is therefore drawing increasing attention.

**Table 1.** Key concentration parameters and cancer therapeutic mechanisms of gases.

Gas	Aqueous solubility (RT, 1 atm)	Cancer therapeutic concentration	Blood poisoning concentration	Anticancer mechanisms

NO	1.8 mM	≥nM levelMost experiments used μM level	25 ppm gas inhalation concentration	Gasotransmitter, inhibits the mitochondrial respiratory metabolism of cancer cells by solidly binding to haem iron centres of many proteinsImpairs cellular bioenergetics after conversion to peroxynitrite
CO	0.93 mM	≥nM levelMost experiments used μM level	100 ppm gas inhalation concentrationCarboxyhaemoglobin level ≥10%30 nM (0.84 ppm) in heart tissue	Gasotransmitter similar to NO, inhibits the mitochondrial respiratory metabolism of cancer cells by solidly binding to haem iron centres of many proteins
H_2_S	0.11 M	≥nM levelMost experiments used μM level	10 ppm gas inhalation concentration	Gasotransmitter similar to NO, inhibits the mitochondrial respiratory metabolism of cancer cells by solidly binding to haem iron centres of many proteinsBe toxic by forming persulfides and polysulphides
H_2_	0.8 mM	Works in the nM∼mM level but the lowest effective concentration is unclear	No obvious toxicity in spite of high concentration	Induces oxidation stress and ROS production
O_2_	0.26 mM	Most experiments used μM∼mM level as hypoxic fraction in tumor ranges from 10 to 30%	Highly biocompatible but toxic at the partial pressure of oxygen >0.5 atm	Blocks tumor angiogenesis and metastasis by abrogating hypoxia in the TME
CO_2_	34 mM	Above solubility to form gas bubbles for assisting imaging and drug release	Toxic under the explosion of air with high CO_2_ concentration (>5%)	No visible therapeutic function
SO_2_	1.47 M	Works in the mM level but the lowest effective concentration is unclear	Negligible haemolytic activity (haemolysis <4%) at 15.8 mg/L SO_2_	Does oxidative damage to tissues/cellsDepletes intratumoral over-expressed GSH, generating oxidative stress

Compared with NO, CO and H_2_S gases, H_2_ has similar anticancer functions and mechanisms, but is much safer because there is no blood poisoning risk at high concentrations and no pro-tumor effects in spite of hydrogen concentration (Table 1). O_2_, SO_2_ and CO_2_ have different anticancer mechanisms. The intratumoral delivery of O_2_ mainly aims to abrogate hypoxia in the TME for blockage of tumor angiogenesis and metastasis, and also to sensitize other oxygen-mediated therapies such as photodynamic therapy and radiotherapy (Table 1). CO_2_ gas has no obvious therapeutic effect but is usually used to mediate contrast-enhanced ultrasound imaging of tumor and controlled release of therapeutic agents (Table 1). In addition, NO and SO_2_ gases can cause oxidative damage to various organelles of cancer cells and deplete intratumoral over-expressed glutathione (GSH), generating oxidative stress (Table 1). In general, most therapeutic gases (NO, CO, H_2_S, H_2_, and O_2_) can sensitize and enhance other traditional therapy methods and also reduce the toxic side effects of traditional therapies.

Therapeutic gases are aimlessly diffusible everywhere in the body, and it is difficult for them to effectively accumulate in target tissues, leading to limited gas therapy efficacy and even potential blood poisoning risk for NO, CO and H_2_S. Therefore, gas-releasing nanomedicines with controlled size and suitable surface properties are being developed to achieve tumor-targeted delivery of gases by integrating gases or gas-releasing molecules (GRMs, or termed gas prodrugs) with nanocarriers by active and passive targeting routes. Moreover, a range of stimuli-responsive GRMs has been developed to realize controlled gas release for enhanced efficacy of gas therapy, which is also assisted by multifunctional nanocarriers. Furthermore, versatile nanomedicines based on multifunctional nanocarriers provide a platform for combination of gas therapy with other traditional therapy modes such as chemotherapy and radiotherapy.

**Figure 1.**
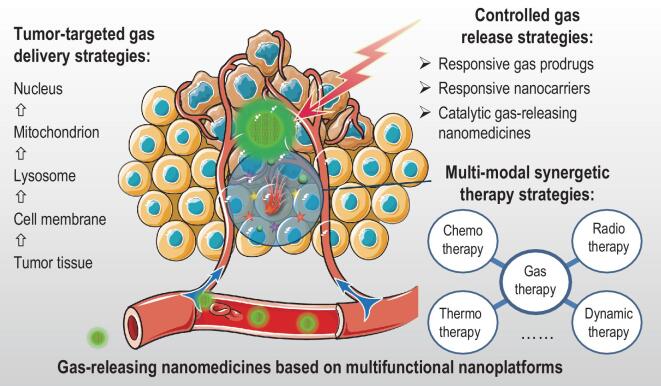
Strategies for engineering advanced nanomedicines for augmented gas therapy of cancer.

Based on the important roles of nanomedicines in gas therapy, this review proposes a series of strategies for engineering advanced nanomedicines for augmented gas therapy of cancer from the following four aspects: (1) stimuli-responsive strategies for controlled gas release; (2) catalytic strategies for controlled gas release; (3) tumor-targeted gas delivery strategies; and (4) multi-model combination strategies based on gas therapy (Fig. 1).

Two stimuli-responsive strategies are introduced for controlled gas release from the view of nanomedicine structure, including engineering of stimuli-responsive prodrugs and stimuli-responsive nanocarriers. Responsive prodrugs and nanocarriers are classed in terms of stimuli sources including external and internal stimuli. We then summarize three types of catalytic strategies for constructing catalytic gas-releasing nanomedicines from the viewpoint of catalytic methods, including photocatalysis, chemical catalysis and enzyme catalysis. We propose a series of tumor-targeted gas delivery strategies for transport nanomedicine, from blood to tumor tissue to tumor cell to intracellular lysosome and then to mitochondrion and nucleus, as well as external magnet-guided delivery. Various combination strategies for enhancing therapy outcome are summarized, by integrating gas therapy with other therapy modes, including chemotherapy, radiotherapy, photothermal therapy, photodynamic therapy and sonodynamic therapy, into a single nanomedicine. Finally, the potential inspiration and application of proposed engineering strategies for gas-releasing nanomedicine development are envisaged. The current trends and future prospects on advanced nanomedicines for gas therapy of cancer are also envisaged. The technical challenges and difficulties are also discussed.

## STRATEGIES FOR ENGINEERING STIMULI-RESPONSIVE GAS-RELEASING NANOMEDICINES

Controlled release of therapeutic gases at the tumor site is one of important routes to enhance gas therapy efficacy and to avoid potential blood poisoning from CO, NO and H_2_S. Here, we summarize two effective strategies for controlled gas release: stimuli-responsive gas-releasing nanomedicines and catalytic gas generation. In terms of nanomedicine structure, stimuli-responsive nanomedicines could be designed by developing responsive GRMs and/or responsive nanocarriers. We review strategies for engineering stimuli-responsive prodrugs and nanocarriers for construction of gas-releasing nanomedicines.

### Engineering of stimuli-responsive GRMs for construction of gas-releasing nanomedicines

Stimuli sources can be divided into two types: exogenous and endogenous stimuli. Exogenous stimuli include light, X-ray, ultrasound, magnet field and heat, while over-expressed chemicals in the TME including H_2_O_2_, lactic acid, glucose and enzymes can be used as endogenous stimuli for stimuli-responsive gas release. Exogenous stimuli-responsive release is easy to manipulate externally, and it is also easy to control the rate and amount of release by adjusting the power and irradiation time of exogenous stimuli. By comparison, endogenous stimuli-responsive release is not limited to tissue depth. Both exogenous and endogenous stimuli-responsive strategies have been widely used to design responsive GRMs and nanocarriers. The following two sections describe strategies for designing exogenous and endogenous stimuli-responsive GRMs and nanocarriers by taking some typical examples.

#### Exogenous stimuli-responsive GRMs for nanomedicines

**Figure 2.**
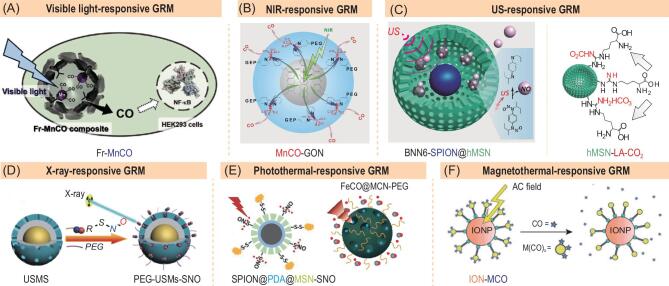
Typical gas-releasing nanomedicines constructed with exogenous stimuli-responsive GRMs. (A) Visible light-responsive Fr-MnCO nanomedicine for controlled CO release. Adapted with permission from [5]. (B) NIR light-responsive MnCO-GO nanomedicine for controlled CO release. Reprinted with permission from [7]. (C) US-responsive BNN6‐SPION@hMSN (left) and hMSN-LA-CO_2_ (right) nanomedicines for controlled NO and CO_2_ release, respectively. Reproduced with permission from [8] and [9]. (D) X-ray-responsive PEG-USMSs-SNO nanomedicine for controlled NO release. Reproduced with permission from [10]. (E) NIR photothermal-responsive SPION@PDA@MSN-SNO and FeCO@MCN-PEG nanomedicines for controlled NO/CO release, respectively. Reproduced with permission from [11] and [12]. (F) Magnetothermal-responsive IONP-MCO nanomedicine for controlled CO release. Reprinted with permission from [13].

Light stimuli-responsive GRMs have attracted great interest for their high spatiotemporal resolution and non-invasive nature. The optical focus enables accurate location and facile control to light. Metal carbonyls (MeCO) are a type of general CO donors (GRMs), which are stabilized by coordination attraction between transition metals and carbonyls and thus are sensitive to UV and/or visible light for photochemical degradation into CO. The coordination feature of MeCO-type GRMs can be used to conjugate them onto functional nanocarriers which are able to coordinate with transition metal ions. In addition, some excellent nanocarriers can be used to improve the dispersion and biocompatibility of GRMs as well as their bioavailability. According to this principle, T. Ueno *et al.* used biocompatible ferritin to coordinate and load manganese and ruthenium carbonyl complexes (MnCO and RuCO) [5,6]. They found that the MnCO-loaded ferritin nanomedicine (Fr-MnCO) maintained the UV/visible light responsiveness of MnCO (Fig. 2A). The amount of CO release was well regulated by light irradiation intensity and time [5]. Ferritin is an excellent nanocarrier with high biocompatibility, small size, large cavity for drug loading and plentiful surface groups which can be used for coordination with many transition metal ions. Therefore, it is especially useful for loading and delivery of transition metal-contained prodrugs such as MeCO. In addition, the active spots inside the internal surface of ferritin can be used for *in situ* growth of various size-controllable metal-contained functional nanoparticles, for example, CuS and Fe_3_O_4_, useful for the integration of imaging with therapy.

However, most GRMs are only sensitive to UV and visible light, leading to limited therapy depth and high phototoxicity. By comparison, the use of near-infrared (NIR) light as a stimulus to design NIR-responsive nanomedicine undoubtedly provides a better choice because of higher tissue penetrability and lower phototoxicity. In this review, we propose two strategies for design and construction of NIR-responsive gas-releasing nanomedicines, including direct synthesis of NIR-responsive GRMs and use of photochemical energy conversion between NIR-absorbed nanocarriers and GRMs for NIR-responsive gas release. Based on the strategy, we constructed MnCO-coordinated and *bis-N*-nitroso compounds (BNN)-stacked graphene oxide nanomedicines (MnCO-GON and BNN-GON) by conjugation of MnCO onto bipyridyl-modified graphene oxide nanosheet (GON) and by π−π stacking between BNN and GON, respectively (Fig. 2B) [7,14]. By virtue of the NIR absorption and photoelectronic characteristics of GON and the photochemical energy conversion between GON and MnCO/BNN, CO/NO were released from the MnCO-GON/BNN-GON nanomedicines under NIR light irradiation with good responsiveness and high ability for controlled release. Besides GON, other NIR-absorbed nanocarriers with GRM-bonded capacity such as black phosphorus nanosheet (BPN) and metal borides could be exploited in future for NIR-responsive gas release. In addition, the NIR-photothermal effect of GON and BPN could also be developed for combination of photothermal therapy with gas therapy.

Ultrasound (US) has high tissue penetration depth (up to 20 cm deep, 1 MHz US wave) and can easily be focused on a small area of the body. Most US-controlled gas-releasing nanomedicines were constructed by encapsulating gas into liposome, and gas release was achieved through the US destruction of liposome. Low gas-loading capacity, poor structural stability, easy gas leakage and one-time/unrepeatable gas release have restricted further application of liposome-based nanomedicines. One solution is encapsulation of US-responsive GRMs into hollow mesoporous silica nanoparticles (hMSN) to construct an US-responsive gas-releasing nanomedicine. It was found that US can stimulate decomposition of *N,N'*-di-*sec*-butyl-*N,N'*-dinitroso-1,4-phenylenediamine (BNN6) into NO free radical, and can also accelerate decomposition of carbonate into CO_2_ gas under intratumoral acidic conditions by the ultrasound cavitation effect (Fig. 2C). Therefore, BNN6 and carbonate-adsorbed *L*-arginine (LA-CO_2_) were encapsulated into hMSN to construct two kinds of US-responsive nanomedicines, realizing US-responsive NO and CO_2_ release, respectively [8,9]. Such nanomedicines demonstrated high gas release controllability and repeatability. Furthermore, low intensity US (1.0 W/cm^2^) can cause effective generation and instant explosion of CO_2_ bubbles, inducing immediate necrosis of panc-1 cells and vascular destruction within panc-1 tumors and thus inhibiting growth of panc-1 tumor. Long-term stabilization of gases within nanocarriers is challenging, but it is much easier for solid GRMs compared with gases. Moreover, triggered gas release from gas-encapsulated nanomedicine is often one-off/unrepeatable, whereas that from GRMs-loaded nanomedicine is repeatable multiple times. The relatively higher stability of hMSN also plays an important role in avoiding leakage of gas prodrugs. In addition, hMSN is a versatile theranostic platform with high specific surface area, adjustable pore size and large central cavity in great support of loading/encapsulating large amounts of various agents including hydrophilic and hydrophobic drugs, imaging molecules/nanoparticles, genes and proteins.

X-ray has high tissue penetration capability and can be used for controlled gas release. We discovered that *S*-nitrosothiols (SNO) were sensitive to X-ray for decomposition into NO. Therefore, an X-ray-responsive nanomedicine was constructed by loading SNO into MSN to successfully realize X-ray-responsive NO release *in vivo* (Fig. 2D) [10]. However, the mechanism of X-ray-triggered NO release from SNO is unclear. X-ray-sensitive GRMs are rarely reported and should be exploited, and their mechanisms for X-ray-responsive gas release are also worthy of in-depth research. In addition, radiotherapy can be easily integrated with gas therapy because both are based on X-ray irradiation.

Some GRMs are relatively stable at normal body temperature but sensitive to high heat for thermal decomposition, such as SNO and MeCO. Therefore, thermally responsive gas release is possibly chased by combination of thermal-sensitive GRMs and stimuli-responsive heat-generating nanocarriers, such as polydopamine (PDA) nanoparticle, mesoporous carbon nanoparticle (MCN) and iron oxide nanoparticle (ION). Light and magnetic fields are two of the most useful exogenous stimuli sources for heat generation. The constructed PDA@MSN-SNO and FeCO@MCN-PEG nanomedicines showed NIR-photothermal-responsive NO and CO release behaviours because of the NIR-photothermal effect of PDA and MCN platforms (Fig. 2E) [11,12]. Similarly, the ION-MCO nanomedicine achieved magnetothermal-responsive CO release from the magnetothermal effect of ION (Fig. 2F) [13]. Such a strategy using thermal energy conversion between stimuli-responsive heat-generating nanocarriers and thermal-sensitive GRMs provides a good route for engineering and constructing thermal-responsive gas-releasing nanomedicines.

#### Endogenous stimuli-responsive GRMs for nanomedicines

**Figure 3.**
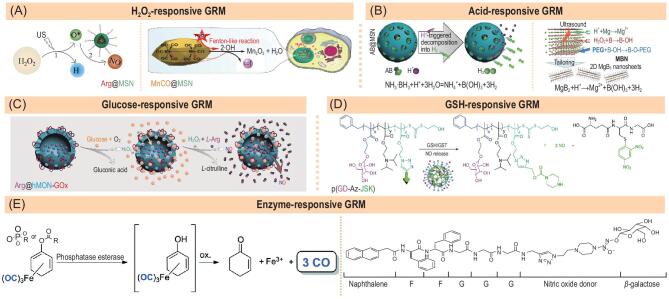
Representative gas-releasing nanomedicines constructed by endogenous stimuli-responsive GRMs. (A) The H_2_O_2_-responsive Arg@MSN and MnCO@MSN nanomedicines for controlled NO and CO release, respectively. Reproduced with permission from [15] and [16]. (B) The acid-responsive AB@MSN and MBN nanomedicines for controlled H_2_ release. Reproduced with permission from [18] and [19]. (C) The glucose-responsive Arg@hMON-GOx nanomedicine for controlled NO release. Reproduced with permission from [20]. (D) The GSH-responsive p(GD-Az-JSK)/DOX nanomedicine for controlled NO release. Reproduced with permission from [21]. (E) The enzyme-responsive CO and NO delivery systems. Reproduced with permission from [22] and [23].

The level of H_2_O_2_ in the TME is significantly higher than in normal cells/tissues, which can be used for intratumoral H_2_O_2_-responsive gas release to enhance the efficacy of gas therapy in tumor. Therefore, it is of practical significance to achieve responsive gas release using H_2_O_2_ in the TME. It was found that *L*-arginine (Arg) and MeCO could be oxidized by strong oxidants such as ·OH to generate NO and CO, and H_2_O_2_ can be decomposed into ·OH through US stimulation and MeCO-mediated Fenton-like reaction. Therefore, Arg@hMSN and MnCO@hMSN nanomedicines were constructed to realize H_2_O_2_-responsive NO and CO release, respectively (Fig. 3A) [15,16]. High H_2_O_2_-responsiveness enabled the nanomedicines to release NO/CO in H_2_O_2_-over-expressed cancer cells rather than in normal cells, exhibiting high anticancer selectivity. It should be noted that the level of H_2_O_2_ in the TME is still limited (generally < 20 μM) and is also easily exhausted during reaction with gas prodrugs, probably limiting the amount of gas production. However, on the other hand, depletion of intratumoral H_2_O_2_ can also induce apoptosis of tumor cells because H_2_O_2_ is a necessary signalling molecule in tumor mediating the pro-survival and pro-proliferative pathways and the metabolic adaption of tumor cells to the TME [17]. Therefore, the anticancer effect of H_2_O_2_-responsive gas-releasing nanomedicines can result from two aspects, gas generation and H_2_O_2_ depletion, which was rarely recognized before.

Weak acidity (pH = 6.5−6.8) in the TME, which is caused by a large proportion of anaerobic glycolysis, is an important characteristic of malignant tumors, and therefore this difference in pH value can be used as a stimulus to design responsive nanomedicines. Ammonia borane (AB) is a hydrogen prodrug with superhigh hydrogen content but poor stability. By amounts of hydrogen bonding interaction between AB and MSN, AB was loaded and stabilized within MSN (AB@MSN) with a superhigh hydrogen loading capacity (130.6 mg H_2_/g MSN), 1370 times higher than that of traditional H_2_ gas@liposome nanomedicine [18]. The constructed AB@MSN nanomedicine can release H_2_ in response to the intratumoral acidic environment (Fig. 3B left). Similarly, Zhang *et al.* used polydopamine (PDA) nanoparticles to encapsulate and stabilize AB through the attraction of hydrogen bonding, and to integrate H_2_ therapy with photothermal therapy (PTT) by virtue of the excellent photothermal effect of PDA [24]. Moreover, we developed a new ultrasound-assisted chemical etching method to synthesize MgB_2_ nanosheet (MBN) and found that MBN can be decomposed by acid to generate H_2_ (Fig. 3B right) [19]. We have discovered that direct nanocrystallization of GRMs is another strategy to construct gas-releasing nanomedicines. In addition, strong gastric acid can be used as an acidic stimulus source for oral administration of acid-responsive gas-releasing nanomedicines for gastric cancer therapy because gas diffusion from the stomach to gastric tumor is relatively quick and easy.

Glucose is a main nutrient and energy source for tumor growth, and tumor consumes more glucose than normal tissues to maintain its rapid growth. Therefore, glucose is often used as an endogenous stimulus for controlled drug release. The content of H_2_O_2_ in the TEM is limited, but glucose oxidase (GOx) can catalyse oxidation of glucose to produce gluconic acid and H_2_O_2_, which could further oxidize Arg to generate NO. Based on this principle, Fan *et al.* constructed a glucose-responsive Arg@hMON-GOx nanomedicine by co-loading GOx and Arg into hollow mesoporous organic silica nanoparticles (hMON) for controlled NO release, as shown in Fig. 3C [20]. The results showed that higher intracellular glucose concentration caused more NO release, and thus a more significant anti-tumor effect. This nanomedicine had high responsiveness to glucose for NO release but poor ability for controlled release. The design and development of novel glucose-responsive gas-releasing nanomedicine with higher performance deserve further study.

GSH is over-expressed and accumulated in the TME and therefore can be used as endogenous stimulus source to control gas release. Meanwhile, glutathione-*S*-transferase (GST) is also selectively over-expressed in some cancer cells such as glioma, and mainly catalyses the covalent reaction *in vivo* between many chemicals and GSH. Therefore, GSH and GST are often combined to trigger controlled drug release for cancer therapy. JSK (*O*^2^-(2,4-dinitrophenyl)-1-[(4-ethoxycarbonyl)piperazin-1-yl]diazen-1-ium-1,2-diolate) is a typically GSH/GST-responsive NO prodrug, which can react with GSH under catalysis of GST to yield NO. Kong *et al.* conjugated JSK onto a copolymer that could self-assemble into nanoparticles, realizing responsive NO release in HepG2 cells (Fig. 3D) [21]. A number of GSH-responsive gas prodrugs have been developed but their nanoformulations are rarely reported. As the intratumoral level of GSH (mM level) is remarkably higher than that of H_2_O_2_ (μM level), GSH should be a plentiful stimulus source to trigger generation of enough gas for cancer therapy. Therefore, development of GSH-responsive gas-releasing nanomedicines is promising.

Some enzymes over-expressed in tumor can also be used as endogenous stimuli to trigger gas release from responsive nanomedicines. Release of gases is achieved using different types or concentrations of enzymes. Carbonxyloxy-/phosphoryloxy-substituted (η^4^-cyclohexadiene)Fe(CO)_3_ complexes reacted with esterase/phosphatase to generate a (dienol)Fe(CO)_3_ intermediate, which was further oxidized to produce CO (Fig. 3E left) [22]. Such an esterase-/phosphatase-responsive strategy has been reported for controlled CO release. However, neither esterase nor phosphatase is specific in the TME, and the consecutive reactions require oxidative conditions, which are also lacking in the TME. Zhao *et al.* caged unstable diazeniumdiolate (NONOate) with glucose and hydrogel to form a stable galactosyl-caged NONOate hydrogel, which can be decoded by galactosidase for responsive NO release (Fig. 3E right) [23]. However, galactosidase is not specifically expressed in tumor, and therefore they further designed a specific galactose, which is indigestible in the body but can be digested by the designed galactosidase, to cage/stabilize NONOate and developed a ‘bump-and-hole’ strategy to realize customized galactosidase-controlled NO release [25]. Such a ‘bump-and-hole’ strategy provides a new route for enzyme-responsive gas release. In addition, there are many enzymes such as matrix metalloproteases (MMPs) over-expressed in the tumor, which can be used to trigger gas release. Design of enzyme-responsive gas-releasing nanomedicines is worth further attention.

### Engineering of stimuli-responsive nanocarriers for construction of gas-releasing nanomedicines

Most GRMs are either easy to spontaneously decompose or only sensitive to some unfavourable stimuli sources such as UV/visible light and high heat, causing limited gas release controllability and bioavailability. Nanocarriers can be designed to be responsive to stimuli for controlled gas release, which could shield the deficit of GRMs. Ideal stimuli-responsive nanocarriers should be engineered to be destructible, degradable, decomposable or convertible. Destructible and degradable nanocarriers can be used to encapsulate spontaneously decomposable GRMs, while convertible nanocarriers can transfer the carrier-absorbing energy to sensitive GRMs for indirectly responsive gas release. Multifunctional nanomaterials play a vitally important role in construction of gas-releasing nanomedicines based on stimuli-responsive nanocarriers. Engineering strategies of destructible/convertible nanocarriers for construction of stimuli-responsive gas-releasing nanomedicines are introduced according to the type of stimuli source.

#### Exogenous stimuli-responsive destructible/convertible nanocarriers for construction of gas-releasing nanomedicines

**Figure 4.**
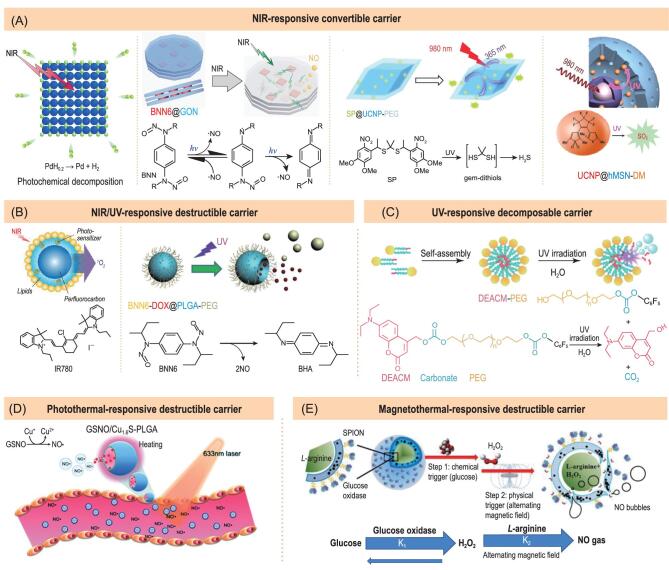
Representative gas-releasing nanomedicines constructed by exogenous stimuli-responsive breakable/convertible carriers. (A) NIR-responsive PdH, BNN6@GON, SP@UCNP-PEG and UCNP@hMSN-DM nanomedicines for controlled release of H_2_, NO, H_2_S and SO_2_, respectively. Reproduced with permission from [14,26–28]. (B) NIR-/UV-responsive breakable nanomedicines for controlled release of ^1^O_2_ and NO. Reproduced with permission from [29] and [30]. (C) UV-responsive decomposable DEACM-PEG nanomedicine for controlled release of CO_2_. Reproduced with permission from [31]. (D) Photothermal-responsive breakable GSNO/Cu_1.6_S-PLGA nanomedicine for controlled release of NO. Reproduced with permission from [32]. (E) Magnetic-thermal breakable nanomedicine for controlled release of NO. Reproduced with permission from [33].

Among various light sources, NIR is most desirable to be used as exogenous stimulus. However, most light-sensitive GRMs are only sensitive to UV/visible light for photochemical degradation into gases. Fortunately, large numbers of NIR-absorbing nanomaterials have been developed as nanocarriers. The key problem here is how to fill the gap between nanocarrier and GRM. We propose two strategies based on convertible nanocarriers to solve the problem: (1) photochemical energy transfer from NIR-absorbing nanocarrier to gas/GRM and (2) NIR-to-UV-to-chemical energy transfer from upconversion nanocarrier to UV-sensitive GRM. Based on the photochemical energy transfer strategy, we incorporated hydrogen into Pd nanocubes and stacked BNN6 (a NO prodrug) between GONs to form a solid solution PdH structure and a sandwich BNN6@GON structure (Fig. 4A), respectively, realizing NIR-photochemical energy transfer and thus NIR-responsive H_2_ and NO release [14,26]. Based on the NIR-to-UV-to-chemical energy transfer strategy, Liu and Yang *et al.* encapsulated UV-sensitive propane-2,2-diylbis((1-(4,5-dimethoxy-2-nitrophenyl) ethyl)-sulphane) (SP, a H_2_S prodrug) and 1-(2,5-dimethylthien-1,1-dioxide-3-yl)-2-(2,5-dimethylthien-3-yl)-hexafluorocyclopentene (DM, a SO_2_ prodrug) into upconversion nanoparticles (UCNP)-based nanocarriers to achieve NIR-responsive H_2_S and SO_2_ release, respectively (Fig. 4A) [27,28]. In addition to reported GON and UCNP, other functional nanoparticles such as BPN and upconversion dyes could possibly be used to realize NIR-responsive gas release based on the proposed energy transfer strategy. Moreover, some disregarded UV-sensitive GRMs could find new applications.

Besides convertible nanocarriers, destructible and decomposable nanocarriers can also be designed. Enough flexible nanocarrier enables generated insoluble gas to burst its structure in support of gas release, while GRM-conjugated decomposable nanocarrier can generate gas under stimulation while its nanostructure is destroyed in favour of gas release. According to the flexible nanocarrier strategy, lipid nanomicelle and monomethoxy(polyethylene glycol)-poly(lactic-*co*-glycolic acid) (mPEG-PLGA) block copolymer nanoparticles as flexible nanocarriers were developed to encapsulate O_2_-rich perfluorohexane/IR780 and BNN6, realizing NIR-/UV-responsive ^1^O_2_/NO release, respectively (Fig. 4B) [29,30]. Based on the nanocarrier decomposition strategy, (7-diethylaminocoumarin-4-yl)methyl carbonate (DEACM, a CO_2_ prodrug) was conjugated on PEG to self-assemble into a prodrug-encapsulated micelle, the structure of which was destroyed under UV light irradiation, which caused CO_2_ release (Fig. 4C) [31].

In addition to mechanical force from gas expansion, flexible nanocarrier can also be engineered to be destructible by thermal expansion of encapsulated functional nanoparticles, such as photothermal and magnetothermal nanoparticles. According to this strategy, Yeh and Gu *et al.* encapsulated Cu_1.6_S nanoparticles and superparamagnetic iron oxide nanoparticle (SPION) together with GSNO and Arg (two kinds of NO prodrugs) into PLGA and liposome nanocarriers, respectively (Fig. 4D, E) [32,33]. By virtue of the photothermal and magnetothermal effects of Cu_1.6_S and SPION, NIR and alternating magnetic field irradiations induced thermal expansion and destruction of PLGA and liposome nanocarriers, then caused NO prodrug release and decomposition into NO. The design and construction of flexible and destructible nanocarriers are key, and integration of flexible organic nanocarrier with heat-generating inorganic nanoparticle into a single nanoparticle is also important and challenging.

#### Endogenous stimuli-responsive destructible/convertible nanocarriers for construction of gas-releasing nanomedicines

**Figure 5.**
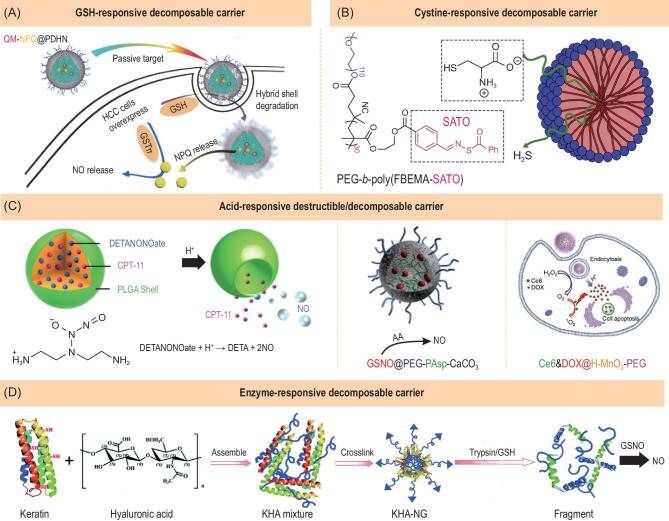
Representative gas-releasing nanomedicines constructed by endogenous stimuli-responsive decomposable/breakable carriers. (A) GSH-responsive decomposable QM-NPQ@PDHN nanomedicine for controlled NO release. Reproduced with permission from [34]. (B) Cystine-responsive decomposable H_2_S-releasing nanomedicine. Reproduced with permission from [37]. (C) Acid-responsive decomposable DETANONOate@PLGA, GSNO@PEG-PAsp-CaCO_3_ and Ce6&DOX@HMnO-PEG nanomedicines for controlled NO and O_2_ release. Reproduced with permission from [38–40]. (D) Enzyme-responsive decomposable KHA-NG nanomedicine for controlled NO release. Reproduced with permission from [41].

Based on the above-mentioned carrier decomposition/destruction strategy, gas nanocarriers can also be engineered to be decomposable/destructible in response to both exogenous and endogenous stimuli. Disulphide bridging is a conventional route to construction of GSH-responsive nanomedicines because intratumoral over-expressed GSH detach the disulphide linkage within the framework of building unit. Li *et al.* constructed a PEGylated disulphide-doped hybrid nanocarrier (PDHN) and loaded NPQ (a hydrophobic NO prodrug) into the nanocarrier (Fig. 5A) [34]. The disulphide-linking shell was decomposed under GSH stimulation in HCC cells, causing release of NPQ (*O*^2^-(2,4-dinitro-5-{[2-(β-d-galactopyranosyl olean-12-en-28-oate-3-yl)-oxy-2-oxoethyl] piperazine-1-yl}phenyl) 1-(methylethanolamino)diazen-1-ium-1,2-dilate) which subsequently decomposed into NO under catalysis of GSTπ. We developed mesoporous organosilica nanoparticles (MON) with a disulphide framework to load 2,4-dinitrobenzenesulphonylchloride (DN, a SO_2_ prodrug) for GSH-responsive carrier degradation and SO_2_ release [35]. Chen *et al*. conjugated *N*-(3-azidopropyl)-2,4-dinitrobenzenesulphonamide (AP-DNs, a SO_2_ prodrug) onto the side chain of methoxy poly(ethylene glycol)-block-poly(*g*-propargyl-*L*-glutamate) (mPEG-PPLG) block copolymer micelle to achieve GSH-responsive decomposition of micelles and sequential release of DN and SO_2_ [36]. With the same strategy, Matson *et al.* constructed a cystine-responsive decomposable micelle carrier by conjugating cystine-responsive *s*-aryl thioxime (SATO, a hydrophobic H_2_S prodrug) onto a block copolymer (Fig. 5B) [37]. Supplementary cystine triggered both the decomposition of SATO and the disassembly of micelle, causing the responsive release of H_2_S. It has not been found that cystine is over-expressed in cancer cells and in the TME, and therefore responsive H_2_S release needs excessive addition of cystine into tumor to trigger.

Based on the above-mentioned carrier destruction strategy, Sung *et al.* used flexible PLGA nanocarrier to encapsulate irinotecan (CPT-11) and acid-responsive diethylenetriamine diazeniumdiolate (DETA NONOate, a NO prodrug) [38]. Intratumoral acid-responsive NO release from DETA NONOate caused destruction of PLGA nanocarrier in support of the co-release of NO and CPT-11 (Fig. 5C left). In addition, amorphous calcium carbonate is highly sensitive to acid, and was therefore used as an acid-decomposable nanocarrier which was stabilized by PEG-PAsp polymer when GSNO was encapsulated (GSNO@PEG-PAsp-CaCO_3_, Fig. 5C centre) [39]. In the acidic TME, acid decomposed/resolved the CaCO_3_-based nanocarrier and subsequently released GSNO, which further reacted with added ascorbic acid (AA) to decompose into NO. Liu *et al.* used hollow MnO_2_ nanoparticle (HMN) as an acid-breakable carrier/prodrug to achieve acid-responsive O_2_ release through carrier decomposition (Fig. 5C right) [40]. Based on the carrier decomposition strategy, a number of stimuli-responsive decomposable nanocarriers such as various metal oxides and phosphates can be developed for construction of gas-releasing nanomedicines.

Expression of specific enzymes such as trypsin and protease-activated receptor-2 (PAR-2) is abnormal in many tumor cells, and is closely related to the biological characteristics and malignancy of tumor cells. Trypsin can cleave the lysine and arginine residues in the polypeptide chain. According to this feature, Li *et al.* constructed a nanogel (KHA-NG) by cross-linking human hair keratin and hyaluronic acid (Fig. 5D) [41]. The KHA-NG was stable under the physiological conditions but could be broken down by trypsin/GSH. The produced fragments stimulated intracellular GSNO to produce NO. In other words, the enzyme-responsive break of constructed nanocarrier finally caused the NO release, realizing the enzyme-responsive NO release. Enzyme-responsive decomposable nanocarriers for gas prodrugs are of value to be developed for gas therapy.

## STRATEGIES FOR CATALYTIC GAS-RELEASING NANOMEDICINES

**Figure 6.**
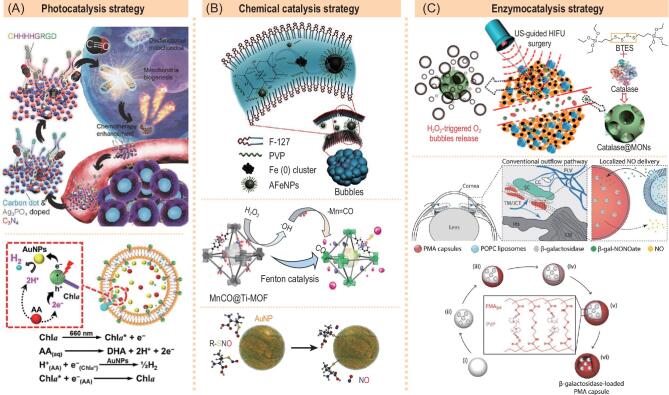
Three catalysis strategies for construction of representative catalytic gas-releasing nanomedicines. (A) Photocatalytic H_2_-releasing HisAgCCN (above) and Chl*α*-AA-AuNPs@liposome (below) nanomedicines. Reprinted with permission from [44] and [45]. (B) Chemically catalytic ROS-releasing Fe nanomedicine (above), CO-releasing MnCO@Ti-MOF nanomedicine (centre) and AuNP nanomedicine (below). Reproduced with permission from [46–48]. (C) Enzymocatalytic catalase@MON (above) and *β*-gal-NONOate@ liposome nanomedicines (below). Reprinted with permission from [49] and [50].

The concept of catalysis originates from chemistry, but has been transplanted to the biomedical field in recent years. Shi and Chen first proposed the concept of catalytic nanomedicine [42,43], opening a window for nanomedicine-mediated gas therapy of cancer. Both high responsiveness and good controllability of gas release are desired, which is different from the main pursuit of high catalysis efficacy in the chemical industry because the effective dose of therapeutic gases is relatively low (nM level or higher). Therefore, the catalytic methodology could be the same or similar, but the standards of screening catalyst and catalysis condition are often different or even opposite between catalytic nanomedicine and industrial catalysis, which provides many important inspirations for nanomedicine. As mentioned above, stimuli-responsive gas release from nanomedicines mainly depends on chemical decomposition of sensitive GRMs and nanocarriers. Such chemical reaction efficiency greatly affects the response rate and the gas release amount. Therefore, to improve gas therapy efficacy by enhancing chemical reaction efficiency, we propose a catalysis strategy for controlled gas release, which is divided into three types, namely photocatalysis, chemical catalysis and enzymocatalysis (Fig. 6). Catalytic gas-releasing nanomedicines can be constructed with only nanocatalyst with endogenous chemicals as substrates, or play a role of catalytic nanoreactor consisting of catalyst and GRM as substrate. Exogenous and endogenous stimulation or combined individual administration of catalyst and GRM can cause catalytic reactions for controlled gas release. These catalysis strategies are introduced in the following sections.

### Photocatalytic generation of therapeutic gases

Photocatalysis can transform light energy into chemical energy using a photosensitive catalyst to accelerate the chemical reaction, and is widely applied in the field of hydrogen evolution. Zheng *et al.* constructed a Z-scheme-type heterogeneous nanocatalyst by co-loading histidine-rich peptide (CHHHHGRGD) and Ag_3_PO_4_ nanoparticles onto the carbon-dot-decorated C_3_N_4_ nanoparticle (HisAgCCN), which was used for photocatalytic reduction of endogenous CO_2_ into CO (Fig. 6A above) [44]. In this work, CO_2_ as one of the catalytic substrates was plentiful in the body, but the loaded histidine-rich peptide as another substrate, reductant and CO_2_ collector was limited, which restricted the amount of CO generation. Moreover, the absorption band of HisAgCCN was located in the UV and visible region, and 630 nm LED light at the power density of 4 W/cm^2^ was used to irradiate tumor-bearing rice for 30 min. Limited tissue penetration depth of visible light and high photothermal effect of irradiated skin tissues affected the outcome of therapy to a certain extent. In the field of solar energy, a broad absorption spectrum of photocatalyst is highly desirable, while strong NIR absorption is purchased by NIR-photocatalytic medicines to obtain high tissue penetration depth and low phototoxicity. Therefore, the development of NIR-photocatalytic gas-releasing medicines is preferred, but still challenging.

By virtue of the photocatalytic hydrolysis strategy, Sung *et al.* prepared a multi-component nanoreactor (NR) where chlorophyll *α* (Chl*α*), *L*-ascorbic acid (*L*-AA) and gold nanoparticles (AuNPs) were co-encapsulated into a liposome nanoparticle (Fig. 6A below) [45]. Zhang and Wu *et al.* also constructed a similar liposome nanoreactor co-encapsulating with Chl*α*, AA and semiconductive polymer dots (Pdots) [51]. These two nanoreactors used the visible light-photocatalytic property of AuNPs and Pdots to realize the photocatalytic generation of H_2_. Similar to the above-mentioned HisAgCCN nanomedicine, these two hydrogen-generating nanomedicines also had drawbacks in the limited amount of sacrificial agent and the irradiation of visible light rather than NIR light. In addition, there was a lack of stability in liposome during hydrogen production [52], which could possibly cause leakage of encapsulated catalyst and substrate. Repeatable on-demand controlled generation of H_2_*in vivo* is worth further investigation.

### Chemically catalytic generation of therapeutic gases

A variety of chemically catalytic and enzymocatalytic reactions occur in the body, including the transport and catalytic oxidation of O_2_ by haem, the enzymocatalytic hydrolysis of protein by metalloproteases, the superoxide dismutase-catalytic disproportion decomposition of O^2−^, the Fe^2+^-catalytic decomposition of H_2_O_2_ into ·OH by the Fenton reaction, the catalase-catalytic decomposition of H_2_O_2_ into O_2_, the NADPH oxidase-catalytic formation of ROS, the GSH reductase-catalytic reduction of GSSH into GSH, *etc*. These necessary catalytic processes ensure the normal function of the body. Such catalytic methods are worth learning about and using to engineer advanced gas-releasing nanomedicines. Chemically catalytic generation of therapeutic gases could improve the responsiveness and efficiency of gas generation.

In the TME, both H_2_O_2_ and acid are highly expressed compared with normal tissues. Therefore, according to the Fenton catalysis strategy, intratumoral delivery of Fenton or Fenton-like catalyst could decompose H_2_O_2_ into highly cytotoxic ·OH for cancer therapy, which is defined as chemodynamic therapy (CDT). Shi *et al.* prepared a kind of amorphous iron (AFe) nanoparticle within the hydrophobic zone of F-127 bilayer, and delivered the nanoparticles as Fenton-like agents to tumor to execute the Fenton catalytic reaction, achieving effective CDT (Fig. 6B above) [47]. Furthermore, ·OH, a strong oxidant generated from the Fenton reaction, can be used as indirect stimulus for controlled gas release. We discovered that MnCO had a dual role as a CO prodrug and a Fenton-like agent, being oxidized by ·OH to generate CO. Therefore, hydrophobic MnCO was encapsulated into hMSN and metal organic framework (MOF) to construct nanomedicines for CO generation by the Fenton catalysis route (Fig. 6B centre) [16,46]. MnCO within the nanomedicines catalysed the decomposition of intratumoral H_2_O_2_ into ·OH, which further oxidized MnCO to release CO, realizing responsive CO release. In normal tissues, lack of Fenton reaction conditions would not lead to decomposition of nanomedicines into CO, causing high tumor selectivity of CO release.

AuNP is a widely applied catalyst, with high surface area and strong capability for coordination with hydrosulphide group. Hervés *et al.* found that AuNP can catalyse low molecular weight of *S*-nitrosothiols (RSNOs) such as *s*-nitrosopenicillamine (SPEN) [48]. Based on the difference in the dissociation energy, the gold−thiol (RS−Au) bond energy is much higher than that of the RS−NO bond, and the S−N bond is therefore easily cleaved by AuNP, favouring formation of RS−AuNP and subsequent release of NO (Fig. 6B below). This catalytic reaction exhibited high efficacy of NO generation. However, how to protect the surface of AuNP from non-specific adsorption of proteins until contact with SNO-type NO prodrug is a question worth considering.

### Enzymocatalytic generation of therapeutic gases

According to the principle of catalase catalysis, intratumoral H_2_O_2_ can be decomposed into O_2_. However, catalase is relatively insufficient in tumor, causing the intratumoral excessive accumulation of H_2_O_2_. Shi *et al.* proposed delivery of catalase to tumor for supplement of O_2_. Based on this strategy, they prepared a catalytic nanoreactor by loading catalase into mesoporous organic silica nanoparticles (catalase@MONs) for tumor-targeted delivery and controlled generation of O_2_ (Fig. 6C above) [49]. This nanoreactor exhibited a high catalytic activity and high sensitivity for the decomposition of H_2_O_2_ even at a low concentration of 10 μM, and it could continuously generate O_2_ gas in a relatively moderate manner, thereby achieving long-lasting contrast enhancement of ultrasound imaging and high efficacy of tumor ablation. Compared with MSN, MON has a faster degradation rate, which is controllable by adjusting the component and amount of incorporated organic/metal compounds, and also exhibits high biocompatibility.

In addition to the use of intratumoral enzymes and substrates as gas prodrugs such as H_2_O_2_, site-targeted individual delivery of specific enzyme and corresponding substrate/GRM is also optional. Stevens *et al.* designed a poly(methacrylic acid) (PMA) capsule to encapsulate *β*-galactosidase, and also prepared a POPC liposome to encapsulate galactose-caged NONOate (*β*-gal-NONOate, a stable NO prodrug) [50]. After reaching the targeted site, the degradation of liposome led to slow leakage of *β*-gal-NONOate, which further catalytically decomposed into NO (Fig. 6C below). High specificity of customized enzyme and substrate promised high selectivity of gas release. Moreover, such a multistep delivery strategy can provide plentiful gas on demand by simply adjusting the administered dosages of enzyme and substrate/GRM. But the activity of biological enzymes is greatly affected by environmental factors, so development of alternative artificial biomimetic enzymes is a direction for future exploration.

## STRATEGIES FOR TUMOR-TARGETED DELIVERY OF GAS-RELEASING NANOMEDICINES

**Figure 7.**
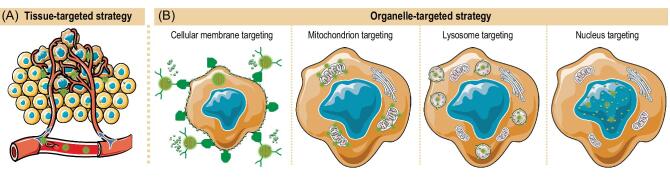
Schematic illustration of the tissue-targeted (A) and organelle-targeted (B) delivery strategies with gas-releasing nanomedicines.

In addition to controlled gas release, tumor-targeted gas delivery is another strategy for augmenting gas therapy efficacy. In this review, the term ‘target’ has two different roles dependent on specific aims, and thus has two different concepts: drug therapeutic target and target for drug delivery. The therapeutic target of NO/CO/H_2_S/H_2_ gases is the mitochondria, and these gases have a specific anti-Warburg function of regulating cellular energy metabolism, consequently exhibiting unique selectivity of killing cancer cells and protecting normal cells from radio/chemotherapeutic damages. On the other hand, the target for drug delivery means the target-mediated (termed ‘targeted’) delivery of drug, and tumor-targeted gas delivery represents the delivery of gas (or its prodrug) towards tumor. The lack of active intratumoral accumulation capability of gases and their small-molecular prodrugs mean that only a small fraction can actually act at the tumor spot after entering the body, which restricts cancer treatment efficacy and might even lead to side effects on normal tissues and blood, especially for highly toxic NO, CO, H_2_S and SO_2_. By virtue of nanotechnology, construction of nanomedicines with tumor-targeting function can resolve this issue. Multitudinous nanomedicines with diverse targeting functions have been developed to realize precision tumor-targeted drug delivery. According to different mechanisms, tumor-targeted delivery strategies can be divided into three types: passive targeting, active targeting and physical targeting. Passive tumor targeting is closely related to intratumoral retention of nanoparticles of suitable size 10−200 nm based on the enhanced permeability and retention (EPR) effect. Active targeting mainly uses tumor-specific targeting agents grafted onto the surface of nanocarriers to recognize tumor tissues/cells/organelles. In addition, physical fields can also be used to guide intratumoral accumulation of functional nanoparticles. Moreover, nanomedicines also favour cellular uptake and intracellular release of gas. The various anticancer mechanisms of gases require different tumor-targeted gas delivery strategies. First tumor tissue-targeting is necessary, which is a prerequisite for further accumulation into tumor cellular organelles including cellular membranes, mitochondria, lysosome and nuclei. Further, tumor cellular organelle targeting could enhance efficiency of gas delivery in favour of high-efficacy gas therapy (Fig. 7).

### Tumor tissue-targeted gas delivery

**Figure 8.**
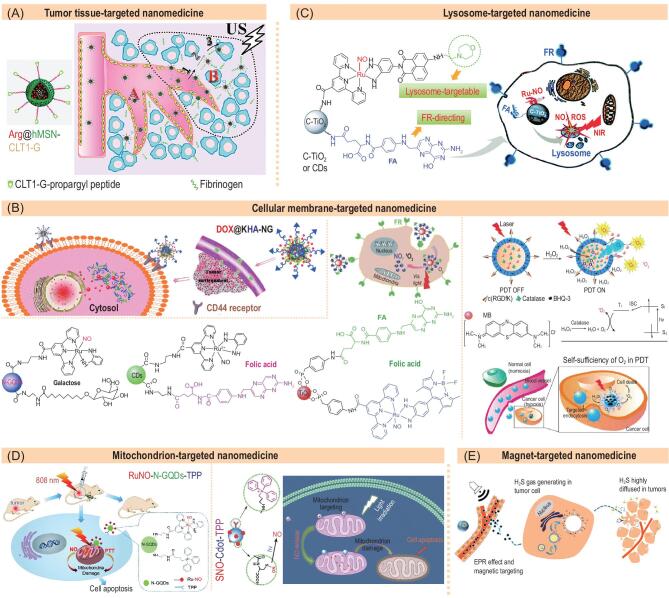
Representative gas-releasing nanomedicines with various targeted functions. (A) Tumor tissue-targeted Arg@hMSN-CLT1-G nanomedicine. Reproduced with permission from [15]. (B) Tumor cellular membrane-targeted DOX@KHA-NGs (left), N-GQDs/CDs/TiO_2_@Gal/FA-RuNO (centre) and O_2_@liposome-RGD (right) nanomedicines. Reproduced with permission from [41,53,54]. (C) Lysosome-targeted C-TiO_2_/CDs@FA-/RuNO-Lyso nanomedicine. Reproduced with permission from [55]. (D) Mitochondrion-targeted RuNO-N-GQD-TPP (left) and SNO-Cdot-TPP (right) nanomedicines. Reproduced with permission from [56] and [57]. (E) Magnet-targeted Fe_2_O_3_-ADT@DPPC-DSPE-PEG_2K_ nanomedicine. Reproduced with permission from [58].

Tumor tissue-targeted delivery of nanomedicine can be achieved by passive intratumoral retention through leaky blood vessels (the EPR effect), active recognition/attachment to tumor blood vessels (such as integrin α_*v*_β_*3*_) and artificially enhanced retention by locally manipulating the nanomedicine size. Based on a retention enhancement strategy, Zhang *et al.* modified Arg-encapsulated MSN nanomedicine (Arg@hMSN-CLT1-G) with cyclic decapeptide (CGLIIQKNEC), which could specifically bind fibrinogen in the extracellular matrix (Fig. 8A) [15]. Such strong binding induced steady retention of the nanomedicine once leaking into tumor, realizing high-efficacy tumor-targeted delivery of NO.

NO is a type of highly reactive molecular free radical, which is able to solidly coordinate with many transition metal ions and directly oxidize DNA, proteins, lipid and GSH. Therefore, the administration/stimulation of excessive NO could induce cellular apoptosis by directly causing oxidative damage to cellular membranes, lysosome and nuclei, and by indirectly inhibiting cellular respiration and energy metabolism through strong coordination with ferroheme. Targeted delivery of NO-releasing nanomedicine to these organelles of cancer cells or to extracellular matrix in the TME could kill cancer cells more effectively.

### Tumor cell membrane-targeted gas delivery

Some specific markers are over-expressed on the membranes of cancer cells, such as integrin α_*v*_β_*3*_, CD44 and receptors of transferrin, folic acid and galactose, the ligands of which could be grafted onto the surface of gas-releasing nanomedicines to recognize cancer cells. Based on this strategy, Liu *et al.* used a variety of multifunctional nanoplatforms (N-doped graphene quantum dots N-GQDs, carbon dots CDs, and TiO_2_ nanoparticles) to co-conjugate RuNO-type NO prodrugs and varied ligands (galactose and folic acid receptors), achieving 4T1 tumor cellular membrane-targeted delivery of NO (Fig. 8B centre) [54]. These inorganic nanocarriers for tumor cell membrane-targeted delivery are easily modified with ligands, and also have various imaging and therapeutic functions in favour of multi-model integration. Similarly, Sun *et al.* incorporated HA (a CD44 ligand) into the KHA-NG nanomedicine to recognize CD44-over-expressed 4T1 cells for tumor cell-targeted NO delivery (Fig. 8B left) [41]. In other typical work, Guo *et al.* modified O_2_-releasing nanomedicine with c(RGDfK) peptide to recognize integrin *α_v_β_3_* over-expressed on U8-MG tumor cells, realizing tumor-targeted O_2_ delivery (Fig. 8B right) [53]. These ligands for tumor cell membrane-targeted delivery enabled more intratumoral accumulation of nanomedicines to a certain extent, but their specificities are not so high. Development of ligands with higher tumor specificity is key to enhance targeted efficacy for gas therapy.

### Lysosome-targeted gas delivery

Lysosome is an important organelle in which nanoparticles are frequently endocytosed. Therefore, lysosome-targeted delivery and release of toxic agents could do damage to lysosome and thus induce cellular apoptosis. High levels of NO, SO_2_ and ROS (especially highly oxidative ·OH and ·O_2_) can cause oxidation damage to the lipid bilayers of tumor cells and their lysosomes. Thus, we proposed a tumor cellular lysosome-targeted NO/SO_2_ delivery strategy for cancer therapy. Based on this strategy, Liu *et al.* conjugated a lysosome-targetable molecule (morpholine) and folic acid (FA) on the surface of NO-releasing nanoplatforms (C-TiO_2_ or CDs@FA-/RuNO-Lyso, Fig. 8C) [55]. This nanoplatform firstly targeted the cellular membrane of FR-over-expressed cancer cells by FA−FR recognition, and then specifically located within the lysosome by the lysosome-targeted morpholine group, where co-release of NO and ROS was triggered by irradiation of NIR light. The lysosome-targeted NO/ROS delivery exhibited outstanding anticancer efficacy compared to non-targeting control groups. In addition, NO can be oxidized by ·O_2_^−^ and stimuli-activable nanocarriers into ONOO^−^ to demonstrate higher oxidation and cytotoxicity for enhanced gas therapy efficacy.

### Mitochondrion-targeted gas delivery

The mitochondrion is the most important target site where many gaseous signalling molecules, including NO, CO, H_2_S and H_2_, have roles in modulating cellular energy to induce cancer cell apoptosis and to protect normal cells from damage. Mitochondrion-targeted delivery of these gases can maximize the efficacy of cancer gas therapy. Some mitochondrion-targeted GRMs had been developed, but they do not prefer to accumulate in tumor cells, causing a large amount of drug loss before arriving at intratumoral cellular mitochondria. The nanomedicine-mediated enhancement of intracellular uptake of gas/GRM favours its further accumulation into/onto intracellular mitochondria. We propose that mitochondrion-targeted gas delivery can be realized by two strategies: (1) grafting of mitochondrion-targetable molecule on the surface of GRM-encapsulated nanocarrier and (2) conjugation of mitochondrion-targeted molecule onto GRM, which is then encapsulated into nanocarrier. Based on the first strategy, Xu *et al.* modified the Cdot nanocarrier with triphenylphosphonium (TPP, a typical mitochondrion-targeted molecule) and visible light-responsive SNO-type NO prodrug to construct a mitochondrion-targeted NO-releasing nanomedicine, which exhibited efficient mitochondria-targeted NO delivery [57]. Similarly, Liu *et al.* also conjugated TPP and RuNO onto the N-GQD nanocarrier (RuNO-N-GQD-TPP) to realize mitochondria-targeted NO delivery [56]. However, the intracellular release of NO from the RuNO-N-GQD-TPP nanomedicine can be stimulated by irradiation of NIR light. It is unclear whether nanomedicines lie on the surface of mitochondria or inside mitochondria, which would possibly affect the mitochondrion-targeted efficacy of released gas. We envisage that gas prodrug is endowed with mitochondrion-targeted function and can more easily enter into mitochondria for high-efficacy mitochondrion-targeted therapy, but this is not yet reported. From tumor tissue to tumor cells to their membranes, to their lysosome and mitochondria, gas-releasing nanomedicines have a very long way to go, especially for mitochondria-targeted gases. Therefore, multistep targeted delivery might be necessary for maximal outcome of targeted gas therapy.

### Magnetic targeting for gas delivery

External fields such as magnetic, ultrasound and electric fields can be used to manipulate nanomedicines to accumulate in tumor, where responsive nanoplatforms play an important role for field control. Liu *et al.* synthesized a magnetic liposome of diameter ∼200 nm by encapsulating anethole dithiolethione (ADT, a hydrophobic H_2_S prodrug) and Fe_2_O_3_ nanoparticles into the hydrophobic shell layer and hydrophilic core of liposome (AMLs), respectively (Fig. 8E) [58]. A high magnetic field induced the transvascular convective movement of AMLs and intratumoral accumulation, enabling intratumoral H_2_S delivery. In addition, magnetic nanoparticles and H_2_S gas could be used for MRI and bubble-assisted ultrasound imaging (USI), respectively, which can be used to monitor tumor-targeted drug delivery. It is worth noting that magnetic field has high tissue penetrability as well as good biocompatibility and various magnetic nanoplatforms are potential candidates, greatly favouring nanomedicine-mediated magnetic targeting for gas delivery. However, precision three-dimensional focalization and manipulation of magnetic field are still challenging and the magnetic responsiveness of existing magnetic nanoplatforms is not high enough, which limits the *in vivo* application of magnetic manipulation to a certain extent.

## COMBINATION STRATEGIES BASED ON GAS THERAPY

**Figure 9.**
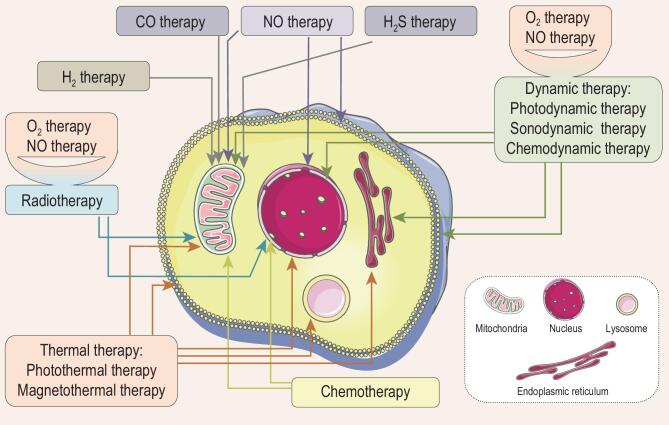
Schematic illustration of target points of various cancer therapy methods.

In recent years, diverse therapy methods including chemotherapy, radiotherapy, thermal therapy, dynamic therapy, immunotherapy, gene therapy and gas therapy have been developed for cancer treatment. The use of a sole treatment mode is inevitably accompanied by some challenges such as side effects, induction of drug resistance and metastasis, and limited therapy efficacy. Combination of different therapy modes, for example, typical integration of chemotherapy and radiotherapy, has been accepted clinically. Gas therapy as an emerging method is found to have remarkably different anticancer mechanisms and targets from other traditional therapy methods, and can be used to assist many other therapy modes to improve therapy effects [51,59]. It is important to understand the roles of gas alone and in combination with other therapies, so here we analyse the targets of most treatment modalities in depth (Fig. 9). CO, H_2_S and H_2_ mainly act on mitochondria, and cause tumor cell apoptosis by disordering mitochondrial functions. Mitochondria, nuclei and membranes of tumor cells can be set as targets for NO. Chemotherapy mainly acts on mitochondria and nuclei, and causes cell death by affecting mitochondrial metabolism and causing damage to nuclei and nuclear substances. The targets of hyperthermia are endoplasmic reticulum, cell membranes, nuclei, lysosome and mitochondria. When combined with radiotherapy, O_2_ and NO act on the nuclei and mitochondria, while when combined with dynamic therapy (photodynamic, acoustic, and chemical dynamic therapies), the organelles acted on are endoplasmic reticulum, cell membranes, nuclei and mitochondria. To make the combined therapy more effective, it is necessary to design a reasonable combination of gas and other treatment methods according to the targets of different treatment modes. Secondly, how to use a single platform to achieve the combined use of different treatment modes and the analysis of their molecular mechanisms is an important and promising direction. Versatile nanomaterials provide a useful platform for integration of varied therapy modes with gas therapy functions into a single particle, accelerating development of multimodal combination therapy of cancer. The following sections summarize a series of engineering strategies for construction of multimodal nanomedicines, with some representative examples.

### Combination of gas therapy and chemotherapy (gasochemotherapy)

Chemotherapy is one of most commonly used cancer treatment methods, but most chemotherapeutic drugs are not specific to cancer cells, leading to inevitable side effects on normal tissues and blood. Chemotherapy also often induces multidrug resistance (MDR) and metastasis, causing the final failure of cancer treatment. It has been found that some gas molecules such as H_2_, NO, CO, H_2_S and SO_2_ can assist chemotherapy for anti-MDR by suppressing the over-expression of MDR-associated proteins, and can also reduce toxic side effects of chemotherapeutic drugs to normal tissues through their normal cell protection effects.

**Figure 10.**
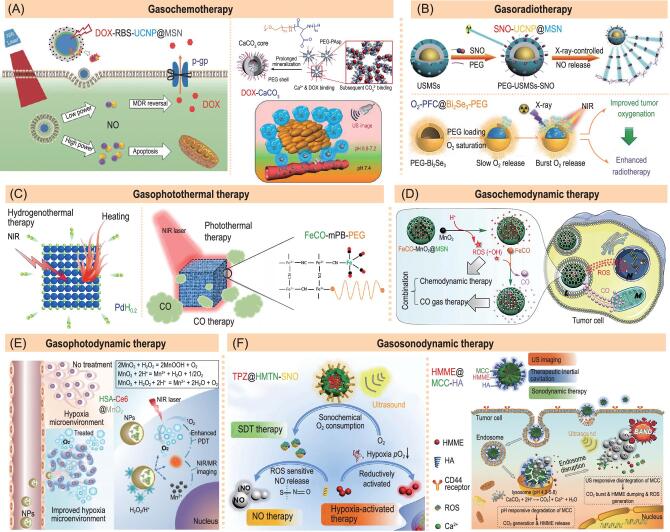
Representative multimodal combination therapy strategies based on gas-releasing nanomedicines. (A) The DOX-RBS-UCNP@MSN (left) and DOX-CaCO_3_ (right) nanomedicines for gasochemotherapy. Reproduced with permission from [62] and [60]. (B) The SNO@UCNP-MSN (above) and O_2_-PFC@Bi_2_Se_3_-PEG (below) nanomedicines for gasoradiotherapy. Reproduced with permission from [10] and [63]. (C) The PdH_0.2_ (left) and FeCO-mPB-PEG (right) nanomedicines for gasophotothermal therapy. Reproduced with permission from [26] and [64]. (D) The FeCO-MnO_2_@MSN nanomedicine for gasochemodynamic therapy. Reproduced with permission from [65]. (E) The HSA-Ce6@MnO_2_ nanomedicine for gasophotodynamic therapy. Reproduced with permission from [66]. (F) The TPZ@HMTN-SNO (left) and HMME@MCC-HA (right) nanomedicines. Reproduced with permission from [67] and [68].

Combination of gas therapy with chemotherapy could solve the issues and challenges of chemotherapy. Min *et al.* encapsulated doxorubicin (DOX) *in situ* into CaCO_3_ nanoparticles to construct a DOX-CaCO_3_ nanomedicine (Fig. 10A right), which achieved intratumoral acid-responsive co-release of CO_2_ gas and DOX, realizing the facile combination of gas therapy and chemotherapy [60]. The CO_2_ bubbles formed during decomposition of CaCO_3_ exhibit USI imaging function and the hollow structure could also be used to load various drugs and act as an excellent theranostic platform. In addition, calcicoptosis from decomposition of CaCO_3_ possibly makes a contribution to combined therapy of tumor [61]. Zhao *et al.* constructed a DOX-RBS-UCNP@MSN nanomedicine by co-encapsulating doxorubicin (DOX, a chemotherapeutic drug) and Roussin's black salt (RBS, a UV-responsive NO prodrug) into MSN nanocarrier (Fig. 10A left), realizing co-release of DOX and NO in cancer cells and consequently overcoming MDR by inhibiting over-expression of P-glycoprotein (P-gp, a drug-transporting protein) [62]. Furthermore, we and Zhang *et al.* proved that combination of CO/NO/H_2_ gas therapy and chemotherapy remarkably reduced side effects of chemotherapy by protecting normal cells and also improved the outcome of cancer therapy, and even restricted the tumor metastasis [19,44].

Toxic side effects of chemotherapy, radiotherapy, thermal therapy and dynamic therapy mainly involve non-specific impairment of off-target drugs to normal tissues, mainly reflected in oxidative damage and inflammation. CO/NO/H_2_S/H_2_ gases are found to have significant anti-inflammation effects at quite low concentrations by activating anti-inflammation signalling pathways of normal cells. As to gas-releasing nanomedicines, cytotoxic therapeutic agents and gas molecules can be gradually and simultaneously leaked from off-target nanomedicines in normal tissues during nanomedicine degradation, where gas can locally play a detoxification role to reduce the toxic side effects of other therapeutic agents. On the other hand, simultaneously released multiple therapeutic agents including gas molecules, all are toxic to varied organelles of cancer cells, with synergistic therapy effects to enhance anticancer outcomes of individual therapy.

### Combination of gas therapy and radiotherapy (gasoradiotherapy)

In addition to chemotherapy, radiotherapy is an important method to treat tumors by damaging DNA in cancer cells. The most obvious limitation of radiotherapy is the radio-resistance caused by hypoxia in the TME. O_2_ and NO as radiosensitizers can capture ionizing radiation-inducing free radicals to form hyperoxide radicals such as ROO·, ONOO^−^ and RNOO, which can stably destroy DNA. However, the intratumoral levels of these two gases are remarkably lower by comparison with normal tissues, with the consequence that cancer cells are not sensitive to ionizing radiation (defined as radio-resistance). Therefore, the most important solution is the supplement of O_2_ and NO to sensitize radiotherapy, bringing about the anticancer strategy of combination of gas therapy and radiotherapy.

X-ray is one of most important ionizing radiation routes to radiotherapy and can also be used for responsive gas release in favour of the facile combination of radiotherapy and gas therapy functions. Based on this strategy, Shi *et al.* constructed SNO-UCNP@MSN nanomedicine by loading X-ray-sensitive SNO into MSN to realize X-ray-responsive release of NO for combination of NO gas therapy and radiotherapy (Fig. 10B above) [10]. MSN is an excellent nanocarrier platform with high surface area for SNO loading, enabling encapsulation of UCNP for theranostic integration. Moreover, Liu *et al.* prepared O_2_-PFC@Bi_2_Se_3_-PEG nanomedicine by encapsulating O_2_-loaded perfluorocarbon (PFC) into hollow Bi_2_Se_3_ nanoparticles (Fig. 10B below), which can not only act as a radio-sensitizer but also release O_2_ under NIR irradiation by the photothermal effect of Bi_2_Se_3_ nanocarrier, overcoming the hypoxia-associated radio-resistance of tumors by a combination of O_2_ gas therapy and radiotherapy [63]. The loading amount of O_2_ was limited because of its low solubility in PFC, and it is necessary to avoid leakage of O_2_.

### Combination of gas therapy and thermal therapy (gasothermal therapy)

Thermal therapy is based on differing thermal tolerance between normal cells (47°C) and cancer cells (42°C) because tumor tissue has a remarkably lower capability of heat dissipation than normal tissues. Hyperpyrexia is able to cause damage to multiple organelles, including cell membranes, mitochondria, nuclei, lysosome and endoplasmic reticulum, inducing cellular apoptosis. To enhance the thermal selectivity to tumor tissue, a number of photothermal and magnetothermal nanomaterials have been developed to specifically accumulate in tumor and *in situ* generate heat intensively by focused irradiation of external light/magnetic fields, even though the irregular thermal dissemination often exceeds the boundary of tumor and damages adjacent normal tissues. Therefore, it is of importance to reduce side effects of thermal therapy. We discovered that some gases such as H_2_, NO and CO which have cellular protection capability, can protect normal cells from non-specific thermal damage [2]. Therefore, we proposed combining gas therapy and thermal therapy into a nanomedicine to avoid the potential side effects of thermal therapy. Based on this strategy, we developed hydrogenated Pd (PdH_0.2_) nanocubes to deliver hydrogen to tumor in a passively targeted and NIR-controlled way and locally generate heat for hydrogenothermal therapy (Fig. 10C) [26]. It was found that H_2_ enhanced efficacy of thermal therapy and also reduced thermal damage to normal cells. Li *et al.* coordinated FeCO within PEGylated mesoporous Prussian blue (mPB-PEG) to construct FeCO-mPB-PEG nanomedicine for NIR-controlled generation of heat and release of CO, achieving a synergy effect of combined CO gas/thermal therapy [64]. Heat-generating functional nanocarriers play an important role in the combination of gas therapy with thermal therapy, and meanwhile thermal-responsive gas prodrugs can also be facilely integrated with such nanocarriers. Research into this is rare, but it is expected there will be development of advanced nanomedicines for gasothermal therapy. In addition, the concept of gasothermal therapy is emerging and could be extended to treatment of other diseases such as bacterial infection and wound healing [69].

### Combination of gas therapy and photo-/sono-/microwave-/chemo-dynamic therapy (gasophoto-/sono-/microwave-/chemo-dynamic therapy)

Photo-/sono-/microwave-dynamic therapy is a type of recently developed cancer treatment, which uses photo-/sono-/microwave-sensitizers to activate O_2_ into singlet oxygen (^1^O_2_) under irradiation of light/sound/microwave, which impairs the organelles of tumor cells including membranes, mitochondria, nuclei and endoplasmic reticulum, and consequently causes their apoptosis. Therefore, the intratumoral supplement of O_2_ by the O_2_-releasing nanomedicines can ameliorate hypoxia in the TME and enhance the efficacy of photo-/sono-/microwave-dynamic therapy of cancer, integrating O_2_ gas therapy and dynamic therapy. Lin *et al.* used MnO_2_ nanoparticles as a carrier of Ce_6_ (a NIR-photosensitizer), as a source of H_2_O_2_ for intratumoral acid-decomposable generation of H_2_O_2_ and as a catalyst for decomposition of H_2_O_2_ into O_2_, realizing efficient O_2_ supplement and high-efficacy NIR-photodynamic therapy for bladder cancer (Fig. 10E) [66]. On the other hand, photo-/sono-/microwave-dynamic reactions can also be used to deprive O_2_ from tumor for oxidation/reduction-triggered drug release. Moreover, in a wide sense of the word, anticancer chemicals generated from photo-/sono-/microwave-/chemo-dynamic reactions involve ROS with no limitation to ^1^O_2_. Feng *et al.* constructed TPZ@HMTN-SNO nanomedicine by loading tirapazamine (TPZ, a reduction-responsive anticancer agent) into hollow mesoporous titanium dioxide nanoparticles (HMTN) with modification of *S*-nitrosothiol (SNO) [67]. Under the irradiation of ultrasound wave, HMTN as sonosensitizer generated ROS for sonodynamic therapy, the generated ROS could decompose SNO into NO for NO gas therapy and the sonodynamic therapy-induced hypoxia further activated TPZ to kill tumor cells (Fig. 10F left). Furthermore, stimuli sources such as light, sound and microwave can also be used to trigger generation of gas and ROS for the combination of gas therapy and dynamic therapy. Zhang *et al.* used the hyaluronic acid (HA) modified mesoporous calcium carbonate (MCC) nanoparticles to load hematoporphyrin monomethyl ether (HMME, a kind of sonosensitizer) to obtain the pH-/ultrasound-responsive HMME@MCC-HA nanomedicine (Fig. 10F right) [68]. Ultrasonication accelerated the intratumoral acid-responsive decomposition of MCC into CO_2_ gas for gas therapy and meanwhile induced the released HMME to generate ROS for sonodynamic therapy, resulting in a synergistic effect of gasosonodynamic therapy. Liu *et al.* constructed NIR-responsive C-TiO_2_@FA-/RuNO-Lyso nanomedicine for NIR-controlled generation of NO and ROS from RuNO and C-TiO_2_, respectively, realizing synergistic gasophotodynamic therapy [55]. In addition to exogenous stimuli sources, endogenous chemicals, especially in the TME, can be used to drive generation of ROS for cancer therapy, defined as chemodynamic therapy. Zhao *et al.* engineered FeCO-MnO_2_@MSN nanomedicine (Fig. 10D) for intratumoral acid-derived sequential release of ROS and CO [65]. A large amount of ·OH was produced from decomposition of MnO_2_ nanoparticles by a Fenton-like reaction for chemodynamic therapy, which then triggered decomposition of co-loaded FeCO into CO for gas therapy, achieving a synergistic effect of chemodynamic therapy and gas therapy. Gasochemodynamic therapy has higher therapeutic depth than gasophotodynamic therapy, and gasochemodynamic therapy is not limited in terms of depth as it uses endogenous TME as stimuli source. It is worth noting that a number of photosensitizers for photodynamic therapy, such as TiO_2_ and porphyrin, can also be developed as sonosensitizers, which provides a good platform for combination of gas therapy and sonodynamic therapy.

## CONCLUSION AND OUTLOOK

Gas therapy is an emerging and promising cancer treatment method, and the development of gas-releasing nanomedicines could provide some special solutions to the issues of cancer treatment. A number of gas-releasing nanomedicines have been developed using multifunctional nanoplatforms, but even so, there are still many gaps between gas therapy and nanomedicines. Here, we have summarized a series of strategies for engineering advanced gas-releasing nanomedicines to solve the issues of gas therapy of cancer, providing guidance for development of more new nanomedicines. Some advanced gas-releasing nanomedicines for cancer treatment reported in recent years are summarized in Table 2. It can be clearly seen that various gases have undergone unbalanced developments. Some gases such as H_2_ and SO_2_ are expected to exhibit their potential in cancer treatment. In addition to cancer, many other inflammation-related diseases including cardiovascular diseases, neurodegenerative diseases and stroke have similar issues with gas therapy. The proposed engineering strategies and developed nanomedicines could also provide inspiration in the treatment of these diseases. Besides the therapy methods involved in this review, some other therapy models such as immunotherapy and gene therapy could be combined with gas therapy by the nanomedicine route to improve cancer treatment outcomes, although these are unreported so far.

**Table 2.** Structure-property relationship of representative gas-releasing nanomedicines for therapy.

Gas	Nanomedicine formulation	Gas loading amount	Gas release efficiency	Trigger for gas release	Theranostic method	Targeted route	Tumor model	Reference

H_2_	AB@MSN	130.6 mg/g	/	Acid	H_2_ therapy	Passive targeting by the EPR effect	4T1 tumor mice	[18]
	AB@PDA nanoparticle	31.9 mg/g	80% (pH = 5, 24 h)	Acid	H_2_ therapy +PTT	Passive targeting by the EPR effect	4T1 tumor mice	[24]
	MBN	173.9 mg/g	100%	Acid	H_2_ therapy +chemotherapy	*In situ* targeting by oral uptake	BGC-823 tumor mice	[19]
	Fe@CMC nanoparticle	19 mg/g	/	Acid	H_2_ therapy PAI	Passive targeting by the EPR effect	4T1 tumor mice	[73]
	PdH_0.2_ nanocube	1.9 mg/g	100%	NIR	H_2_ therapy + PTT+PAI+PTI	Passive targeting by the EPR effect	4T1, B16-F10 tumor mice	[26]
	PdH-MOF nanoparticle	9.4 mg/g	100%	·OH	H_2_ therapy + PTT+PAI+PTI	Passive targeting by the EPR effect	4T1 tumor mice	[74]
	Chlα-AA-Au@liposome	/	/	Visible light catalysis	H_2_ therapy	/	Inflammation	[45]
	Pdot-AA@liposome	/	/	Visible light catalytic	H_2_ therapy	/	Inflammation	[51]
	Mg@MSN	79 mg/g	/	Spontaneous release	H_2_ therapy	/	PC12 cells	[75]
	Mg@PLGA microparticle	7 mg/g	/	Spontaneous release	H_2_ therapy	/	Osteoarthritis	[76]
	H_2_-C_3_F_8_@DSPE-PEG microbubble	/	/	Spontaneous release	H_2_ therapy +USI	/	Myocardial ischemia	[52]
CO_2_	hMSN-Arg-CO_2_	50.6 mg/g	/	US	CO_2_ therapy +USI	Passive targeting by the EPR effect	Panc-1 tumor mice	[9]
	DEACM-PEG micelle	/	/	UV	CO_2_ therapy	Passive targeting by the EPR effect	Renca cells	[31]
	DOX-CaCO_3_-Pasp-PEG nanoparticle	/	/	Acid+US	CO_2_ therapy +chemotherapy +USI	Passive targeting by the EPR effect	SCC-7 tumor mice	[60]
	HMME@MCC-HA	333.6 mg/g	93% (pH = 5.8+US, 24 h)	Acid+US	CO_2_ therapy + SDT+USI	Cell membrane targeting	MCF-7 tumor mice	[68]
	CaCO_3_-DOX@PLG-RVG nanoparticle	207.3 mg/g	/	Acid	CO_2_ therapy +chemotherapy +USI	Cell membrane targeting	N2a tumor mice	[77]
	DOX–HCO_3_@hMSN	/	/	Acid	CO_2_ therapy +chemotherapy	/	MCF-7 and MCF-7/ADR cells	[78]
	ABC@HSPC liposome	/	/	Thermal	CO_2_ therapy+USI	/	HT1080 cells	[79]
	ABC-DOX@DSPE-PEG liposome	55.7 mg/g	/	Thermal	CO_2_ therapy +chemotherapy +USI	/	H460 cells	[80]
	ABC-DOX-Au-@liposome-MUC1 aptamer	/	/	NIR-photothermal	CO_2_ therapy +chemotherapy +PTT+USI+FI	Cell membrane targeting	MCF-7 tumor mice	[81]
SO_2_	UCNP@hMSN-DM	15.8 mg/g	/	NIR	SO_2_ therapy	Passive targeting by the EPR effect	S180 tumor mice	[28]
	DOX@DN-PLG-mPEG micelle	3.5 mg/g	90% (120 min)	GSH	SO_2_ therapy +chemotherapy	Passive targeting by the EPR effect	MCF-7 ADR tumor mice	[36]
	MON-DN@PCBMA-DOX	11.4 mg/g	/	GSH	SO_2_ therapy +chemotherapy	Passive targeting by the EPR effect	MCF-7 ADR tumor mice	[35]
O_2_ ROS	O_2_-PFH-IR780@Lipid	/	/	NIR	O_2_ therapy +PDT	Passive targeting by the EPR effect	CT26 tumor mice	[29]
	O_2_-PFC@Bi_2_Se_3_-PEG nanoparticle	3.1 mg/g	/	NIR-photothermal	O_2_ therapy +radiotherapy	Passive targeting by the EPR effect	4T1 tumor mice	[63]
	DOX/CP-NI nanoparticle	/	/	Vis/NIR	PDT +chemotherapy +MRI	Passive targeting by the EPR effect	HeLa tumor mice	[82]
	Ce6-MnO_2_@HSA	/	/	Acid	O_2_ therapy +PDT+MRI+FI	Passive targeting by the EPR effect	MB-49 tumor mice	[66]
	Ce6-DOX@H-MnO_2_-PEG nanoparticle	/	/	Acid	CDT +chemotherapy +immunotherapy +MRI	Passive targeting by the EPR effect	4T1 tumor mice	[40]
	MnO_2_@HA-Man nanoparticle	/	/	H_2_O_2_+acid	O_2_ therapy +MRI	Tumor/TAMs targeting	4T1 tumor mice	[83]
	UCSD@SiO_2_@MnO_2_	/	/	H_2_O_2_+acid	O_2_ therapy +PDT +radiotherapy +FI	Passive targeting by the EPR effect	4T1 tumor mice	[84]
	GOD-Fe_3_O_4_@DMSN	/	/	Fenton catalysis	CDT	Passive targeting by the EPR effect	4T1 and U87 tumor mice	[43]
	AFe nanoparticle	/	/	Fenton catalysis	CDT+MRI	Magnetic targeting	4T1 tumor mice	[47]
	C dots-C_3_N_4_@-PpIX-PEG-RGD nanoparticle	/	1.4 mg/L (630 nm, 20 min)	Photocatalysis	O_2_ therapy+PDT	Tumor targeting	4T1 tumor mice	[85]
	Catalase@MON	/	/	Catalase catalysis	O_2_ therapy + HIFU thermal therapy+USI	Passive targeting by the EPR effect	MB-231 tumor mice	[49]
	Catalase@liposome-RGD	/	/	Catalase catalysis	O_2_ therapy +PDT	Tumor targeting	U87-MG tumor mice	[53]
H_2_S	SP@UCNP-PEG	1.92 × 10^−18 ^g/particle	/	NIR	H_2_S therapy	/	L929 cells	[27]
	DATS@MSN	84.4 mg/g	/	GSH	H_2_S therapy	/	Ischemic/reperfusion injury	[86]
	PEG-*b*-poly(FBEMA-SATO) micelle	/	/	Cystine	H_2_S therapy	/	HCT116 cells	[37]
	HA-JK1 hydrogel	/	/	Acid	H_2_S therapy	/	Cutaneous wound model	[87]
	HS-ASP/PTX@PCL-PEG micelle	4.5 mg/g	88.2% HS-ASP (pH = 5, 24 h)	Acid	H_2_S therapy +chemotherapy	/	LL/2 cells	[88]
	ADT-Fe_2_O_3_@Liposome	/	/	CBS/CSE	H_2_S therapy +USI+MRI	Magnetic targeting	HepG2 tumor mice	[58]
	BSA/ALA/DADS nanoemulsion	/	/	Spontaneous release	H_2_S therapy	/	MCF-7 and HuT 78 cells	[89]
CO	MnCO-GON	881 mg/g	100%	NIR	CO therapy	/	Raw264.7 cells	[7]
	FeCO-mPB-PEG nanoparticle	/	73%	NIR	CO therapy +PTT+USI	Passive targeting by the EPR effect	HeLa tumor mice	[64]
	MnCO-UCNP@PL-PEG	/	91%	NIR	/	/	/	[90]
	FeCO-DOX@MCN-PEG	/	/	NIR-photothermal	CO therapy +PTT +chemotherapy +PAI	Passive targeting by the EPR effect	MCF-7 tumor mice	[12]
	MnCO-Ferritin	/	/	Visible light	CO therapy	/	HEK-293 cells	[5]
	QD-MnCO	/	/	Visible light	CO therapy	/	/	[91]
	MnCO-WTPhC	7.8 mol/mol	96.7%	Visible light	CO therapy	/	HEK293 cells	[92]
	MnCO-nanodiamond	7.1 mg/g	/	UV light	CO therapy	/	/	[93]
	MnCO@Al-MCM-41 nanoparticle	31.5 mg/g	/	UV light	CO therapy	/	/	[94]
	MnCO@SFN	1.2 mg/g	39.3%	UV light	/	/	/	[95]
	MnCO@hMSN	337.4 mg/g	100%	H_2_O_2_	CO therapy	/	4T1 tumor mice	[16]
	MnCO@Ti-MOF	/	/	H_2_O_2_	CO therapy	/	AGS cells	[46]
	IONP-RuCO	/	/	Magnetothermal	CO therapy	/	/	[96]
	IONP-RuCO	28 mg/g	/	Magnetothermal	/	/	/	[13]
	C dots/Ag_3_PO_4_-C_3_N_4_-RGD nanoparticle	/	/	Visible light catalysis	CO therapy +chemotherapy	Tumor targeting	PC-3 tumor mice	[44]
	FeCO-MnO_2_@MSN	118.7 mg/g	100%	Acid	CO therapy+CDT	Passive targeting by the EPR effect	4T1 tumor mice	[65]
	RuCO-DOPA-PBAN	3 mol/mol	40% (10 mM cysteine, 2 h)	Cysteine	CO therapy	/	RAW264.7 macrophages	[97]
	PEG-bI-OrnRu-bI-nBu micelle	22.3 mg/g	/	Cysteine	CO therapy	/	THP-1 Blue cells	[98]
	CORM2@SMA micelle	18.0 mg/g	/	Spontaneous release	CO therapy	/	KG-1 cell	[99]
NO	BNN6‐SPION@hMSN	134.5 mg/g	/	US	NO therapy +MRI	/	HeLa cell	[8]
	TPZ@HMTN-SNO	/	/	US	NO therapy +SDT therapy +hypoxia-activated therapy+USI	EPR effect	MCF-7 tumor mice	[67]
	Arg@hMSN-CLT1-G	29.3 mg/g	/	H_2_O_2_+US	NO therapy	Tumor targeting	Panc-1 tumor mice	[15]
	PEG-USMS-SNO	/	/	X-Ray	NO therapy +radiotherapy	Passive targeting by the EPR effect	4T1 tumor mice	[10]
	BNN6-GON	259 mg/g	100%	NIR	NO therapy	/	143B cells	[14]
	C-TiO_2_@Lyso-RuNO/FA nanoparticle	19.5 mg/g	/	NIR	NO therapy +PDT	Cell membrane-lysosome targeting	HeLa cells	[55]
	N-GQDs@RuNO/TPP	48 mg/g	/	NIR	NO therapy +PTT	Mitochondria targeting	HeLa tumor mice	[56]
	RBS/DOX-UCNP@MSN	39 mg/g	/	NIR	NO therapy +chemotherapy	/	HeLa and MCF-7 cells	[62]
	SPION@PDA@MSN-SNO/DOX	/	/	NIR-photothermal	NO therapy +chemotherapy +FI	Passive targeting by the EPR effect	MCF-7/ADR tumor mice	[11]
	GSNO/Cu_1.6_S@PLGA nanoparticle	/	/	Visible Photothermal	NO therapy +PTT	/	MRC-5 cells	[32]
	RuNO-TiO_2_ nanoparticle	4.8 mg/g	/	Visible light	NO therapy +PDT	Cell membrane targeting	HeLa cells	[54]
	Cdot@SNO/TPP	0.96 mg/g	/	UV Light	NO therapy	Mitochondria targeting	HepG2, A549 and HeLa cells	[57]
	BNN6-DOX@mPEG-PLGA micelle	/	/	UV light	NO therapy +chemotherapy	/	OVCAR-8/ADR cells	[30]
	Arg@MV-GOx	/	/	Magnetothermal +glucose	NO therapy +MRI	Passive targeting by the EPR effect	Diabetic mice	[33]
	DETANONOate-CPT11@PLGA	/	/	Acid	NO therapy +chemotherapy	Passive targeting by the EPR effect	MCF-7/ADR tumor mice	[38]
	GSNO@CaCO_3_-PAsp nanoparticle	11.2 mg/g	85.3% (pH = 5, 24 h)	Acid	NO therapy +chemotherapy	/	MCF-7 cells	[39]
	Arg@hMON-GOx	22.4 mg/g	/	Glucose	NO therapy + starving therapy+USI	Passive targeting by the EPR effect	U87MG tumor mice	[20]
	p(GD-Az-JSK)/DOX	/	58.8%	GSH/GST	NO therapy +chemotherapy	Cell membrane targeting	HepG2 cells	[21]
	DOX@KHA nanogel	/	/	Tyrosine +GSH	NO therapy +chemotherapy	Cell membrane targeting	4T1 tumor mice	[41]
	QM-NPQ@PDHN	/	/	GSH+GSTπ	NO therapy+FI	Passive targeting by the EPR effect	SMMC-7721 tumor mice	[34]
	RSNO-Au nanoparticle	/	100%	Chemical catalysis	/	/	/	[48]
	β-gal-NONOate@PMA capsule	/	/	Biocatalysis	NO therapy	/	Glaucoma	[50]

AA, ascorbic acid; AP-DN, *n*-(3-azidopropyl)-2,4-dinitrobenzenesulphonamide; Arg, *L*-arginine; ALA, α-linolenic acid; ADT, anethole dithiolethione; ASP, aspirin; BSA, bovine serum albumin; CBS, cystathionine-β synthase; CSE, cystathionine-γ-lyase; CPT-11, irinotecan; CDT, chemodynamic therapy; CMC, carboxymethyl cellulose; Chlα, chlorophylla; CP-NI, 2-nitroimidazole-grafted conjugated polymer; DOX, doxorubicin; DSPE-PEG, 1,2-distearoyl-sn-glycero-3-phosphoethanolamine-*N*-[methoxy(polyethylene glycol)]; DETANONOate, diethylenetriamine diazeniumdiolate; DEACM, (7-diethylaminocoumarin-4-yl)methyl; DADS, diallyl disulphide; DM, 1-(2,5-dimethylthien-1,1-dioxide-3-yl)-2-(2,5-dimethylthien-3-yl)-hexafluorocyclopentene; DMSN, dendritic mesoporous silica nanoparticle; EPR effect, enhanced permeability and retention effect; FBEMA, 2-(4-formylbenzoyloxy)ethyl methacrylate; FA, folic acid; FeCO, carbonyl iron compounds; FI, fluorescence imaging; GSH, glutathione; GST, glutathione *S*-transferase; GOD, glucose oxidase; HA, hyaluronic acid; HSPC, hydrogenated soy phosphatidylcholine; HA, hyaluronic acid; HMTN, hollow mesoporous titanium dioxide nanoparticle; HMME, hematoporphyrin monomethyl ether; HIFU, high intensity focused ultrasound; IONP, iron oxide nanoparticle; JSK, *O*^2^-(2,4-dinitrophenyl) 1-[(4-ethoxycarbonyl) piperazin-1yl]diazen-1-ium-1,2-diolate; JK1, hydrogen sulphide donor (C_8_H_8_Li_3_NO_3_PS); KHA, keratin-hyaluronic acid; Lyso, lysosome; MCC, mesoporous calcium carbonate; MRI, magnetic resonance imaging; mPB, mesoporous Prussian blue; MBN, magnesium boride nanosheet; Man, mannan; MV, magnetic microvesicle; NPQ, *O*^2^-(2,4-dinitro-5-{[2-(β-d-galactopyranosyl olean-12-en-28-oate-3-yl)-oxy-2-oxoethyl] piperazine-1-yl}phenyl) 1-(methylethanolamino)diazen-1-ium-1,2-dilate; N-GQDs, N-doped graphene quantum dots; NIR, near-infrared light; PpIX, protoporphyrin; PDHN, PEGylated disulphide-doped hybrid nanocarrier; PTX, paclitaxel; PFH, perfluorohexane; p(GD-Az-JSK), nitric oxide prodrug molecule copolymer; PFC, perfluorocarbon; PCL, polycaprolactone; PAI, photoacoustic imaging; PTI, photothermal imaging; PTT, photothermal therapy; PDT, photodynamic therapy; PEG-bI-OrnRu-bI-nBu, poly(ethylene glycol)-bpoly[Ru(CO)_3_Cl(ornithinate acrylamide)]-*b*-poly(*n*-butylacrylamide); PDA, polydopamine; Pdot, polymer dot; PEG-PAsp, poly(ethylene glycol)-*b*-poly(*L*-aspartic acid) copolymer; PLG, poly(*D, L*-lactide-*co*-glycolide); QM, quinolone-malononitrile derivative; QD, quantum dot; RuCO, ruthenium carbonyl compounds; RGD, arg-gly-asp; RVG, rabies virus glycoprotein; SFN, silk fibroin nanoparticle; SDT, sonodynamic therapy; SPION, superparamagnetic iron oxide nanoparticle; SPCD, silicon phthalocyanine dihydroxide; TPP, triphenylphosphonium; TPZ, tirapazamine; USMS, UCNP@mSiO_2_; UCSD, UCNP&SPCD; US, ultrasound; UV, ultraviolet light; Vis, visible light; WTPhC, wild-type polyhedral crystal.

For stimuli-responsive gas-releasing nanome-dicines, some advanced stimuli sources such as various specific enzymes, microwaves, NIR-II light, electricity and magnet fields have rarely been employed, and many nanocarriers with corresponding functions could be developed in depth for construction of advanced gas-releasing nanomedicines. Most current targeting paths focus only on one or two organelles, and more attention should be paid to a multistep targeting strategies to improve targeted efficiency of nanomedicines. Some gases such as NO and SO_2_ can damage the nuclei of cancer cells, and therefore direct nuclei-targeted delivery of these gases could possibly improve their anticancer efficacies. However, nuclei-targeted gas delivery by nanomedicines has not been reported so far. Catalytic strategies for controlled gas release are emerging, and many strategies in industrial catalysis could be used as reference.

Along with quick development of gas therapy-related basic research, a large number of ‘from bench to beside’ clinical trials have been executed. Several drugs for NO therapy, such as nitroglycerin, sodium nitroprusside, isosorbide mononitrate, hydralazine and 2-nicotinamidoethyl nitrate, have been widely used clinically for urgent treatment of cardiovascular diseases, and their clinical applications are also being extended to cancer treatment. The main adverse effects involve blood poisoning to a certain extent because of poor controllability of NO release and high toxicity of decomposition products. A phase II study of concurrent chemotherapy and radiotherapy with nitroglycerin in advanced non-small cell lung cancer demonstrated an acceptable toxicity profile [70,71]. Recently, phase Ib/II trial results indicated the safety, tolerability and promising activity of NG-monomethyl-*L*-arginine (*L*-NMMA, iNOS inhibitor) in combination with chemotherapy in treatment of chemotherapy-refractory triple negative breast cancer (TNBC) [72]. It implies that besides the intratumoral delivery of therapeutic NO/CO/H_2_S gases and their prodrugs, the tumor-targeted delivery of related iNOS/HO1/CBS inhibitors by nanomedicine approach also holds great promise to improve anticancer efficacy and avoid side effects. In addition, direct inhalation of therapeutic gases such as NO and O_2_ has been widely used in clinic, especially to treat respiratory diseases such as novel corona virus disease (COVID-19), but have a potential poisoning risk from overdose. Compared with other several therapeutic gases, use of H_2_ gas therapy is a recently developed method for treating numerous diseases, but has huge prospects because of the high biosafety and wide-spectrum therapeutic effects of H_2_. More than 60 clinic trials of hydrogen therapy have been reported so far for treatment of various inflammation-related diseases, including assistance of radiotherapy and chemotherapy and oxygen therapy of COVID-19 pneumonia [100], mainly through inhalation of H_2_ gas and oral uptake of hydrogen-rich water. The outcome of hydrogen therapy can be further improved by nanomedicine strategies, especially aiming at deep-seated tumors such as glioma and hepatocarcinoma. Development of H_2_-releasing nanomedicines for cancer therapy is still at the preclinical stage, but is worth further exploration. The existing H_2_ prodrugs for construction of nanomedicines are only few, and the controlled hydrogen release strategy also needs to be extended for development of more H_2_-releasing nanomedicines. Varied routes to hydrogen administration including oral uptake, injection and surface dressing could be exploited, which would create further need for specific nanomedicines. The use of H_2_-releasing nanomedicines for improvement of many therapy modes is still a blank page.
